# Web-Based Information Seeking Behaviors of Low-Literacy Hispanic Survivors of Breast Cancer: Observational Pilot Study

**DOI:** 10.2196/22809

**Published:** 2021-10-27

**Authors:** Francisco Iacobelli, Ginger Dragon, Giselle Mazur, Judith Guitelman

**Affiliations:** 1 Computer Science Department Northeastern Illinois University Chicago, IL United States; 2 ALAS-WINGS Chicago, IL United States

**Keywords:** low literacy, health literacy, online searches, Hispanic breast cancer survivors

## Abstract

**Background:**

Internet searching is a useful tool for seeking health information and one that can benefit low-literacy populations. However, low-literacy Hispanic survivors of breast cancer do not normally search for health information on the web. For them, the process of searching can be frustrating, as frequent mistakes while typing can result in misleading search results lists. Searches using voice (dictation) are preferred by this population; however, even if an appropriate result list is displayed, low-literacy Hispanic women may be challenged in their ability to fully understand any individual article from that list because of the complexity of the writing.

**Objective:**

This observational study aims to explore and describe web-based search behaviors of Hispanic survivors of breast cancer by themselves and with their caregivers, as well as to describe the challenges they face when processing health information on the web.

**Methods:**

We recruited 7 Hispanic female survivors of breast cancer. They had the option to bring a caregiver. Of the 7 women, 3 (43%) did, totaling 10 women. We administered the Health LiTT health literacy test, a demographic survey, and a breast cancer knowledge assessment. Next, we trained the participants to search on the web with either a keyboard or via voice. Then, they had to find information about 3 guided queries and 1 free-form query related to breast cancer. Participants were allowed to search in English or in Spanish. We video and audio recorded the computer activity of all participants and analyzed it.

**Results:**

We found web articles to be written for a grade level of 11.33 in English and 7.15 in Spanish. We also found that most participants preferred searching using voice but struggled with this modality. Pausing while searching via voice resulted in incomplete search queries, as it confused the search engine. At other times, background noises were detected and included in the search. We also found that participants formulated overly general queries to broaden the results list hoping to find more specific information. In addition, several participants considered their queries satisfied based on information from the snippets on the result lists alone. Finally, participants who spent more time reviewing articles scored higher on the health literacy test.

**Conclusions:**

Despite the problems of searching using speech, we found a preference for this modality, which suggests a need to avoid potential errors that could appear in written queries. We also found the use of general questions to increase the chances of answers to more specific concerns. Understanding search behaviors and information evaluation strategies for low-literacy Hispanic women survivors of breast cancer is fundamental to designing useful search interfaces that yield relevant and reliable information on the web.

## Introduction

### Background

Internet searching has become an increasingly popular tool for patients to find health-related information [1], and it has been linked to improved health outcomes and greater patient engagement [2]. Despite efforts to mitigate the challenges that Hispanics face when seeking information on the web (guided user searches [3] and video, audio, and simplified text [4-7]), Hispanics still do not use web-based health information at the same rate as non-Hispanic White individuals do.

In this paper, we report an observational study that explores web-based information seeking behaviors (search and selection of results) of low-literacy Hispanic survivors of breast cancer and some of their caregivers when using either a voice- or text-based search engine. We describe behaviors that stress the difficulty of processing web-based information by this population as well as attributes inherent to the interface (traditional search engine or voice search engine) that make this task even more difficult and provide recommendations for future search interface designs.

### Health Information Search Behaviors and Hispanics

The Health Information National Trend Survey has shown for several years that the internet is the most used source of health information [8]. Searching for health information on the web has been linked with improved health outcomes and greater patient engagement [2]. A study on US adults found that using the internet for health information has been strongly correlated with self-reports of very good or excellent health status, and the largest increase in health status has been observed in adults without a high school diploma [9]. However, education is also highly correlated with the kind of information favored by individuals. For example, adults with a high school diploma use more text-based sources (the dominant modality of web-based sources), whereas adults without a high school diploma use more verbal sources [9]. Consistent with this, another study of US adults’ trends over 4 years revealed that education level was positively correlated with using the internet for health information and negatively correlated with using friends, family, and coworkers for health information [8].

Although this suggests that populations with lower literacy should benefit the most from web-based health information seeking, they do not search for web-based health information frequently.

As health information migrates to digital formats, Hispanics and other minorities are at a disadvantage, as more of them do not report using the internet as their source of information (except through surrogates) [10]. In particular, researchers found that in recent years (2011-2016), US-born Mexicans and foreign-born Hispanics do not use the internet for health information seeking or for sending emails to health providers as US-born non-Hispanic White people do [11,12]. Overall, Hispanic participants are more likely to use health care professionals as a source of health information compared with non-Hispanic participants. Moreover, being older, having low internet skills, and being Hispanic were determinants of using a health care provider or traditional media, such as print and magazines, as a source of health information versus using the web. Being Hispanic and having a history of cancer is highly correlated with using health care professionals as a primary source of health information [8]. These studies suggest that Hispanic survivors of breast cancer do not use the internet to find information.

### Barriers to Accessing Web-Based Health Information

Hispanics of low socioeconomic status may be at an even further disadvantage of using web-based health information. Research that has tried to explain the barriers that low socioeconomic status individuals may encounter while trying to seek health information on the web has found that spotty internet access as well as frustration with the information search process are detrimental to seeking information on the web [13]. Web-based searching is a challenging task for low-literacy Hispanics. Over a decade ago, Birru et al [14] described problems with formulating queries, selecting and understanding results by low-literacy Hispanics who searched for information independently. As to what mode of internet search is favored by low-literacy adults, they tend to prefer voice searching (dictating search queries) to written searches when given the option [15]. This can be a strategy to mitigate common mistakes such as misspelling, misappropriation of words (writing lymphoma when they mean lymphedema), and incomplete search queries that can result in inadequate results and misleading information [14].

In addition, the complexity of information on the web is difficult to process for individuals with low literacy. Most internet health content is written at a level that is above the average reading level of adults in the United States [16-20]. For example, Walsh and Volsko [19] analyzed the reading levels of 100 publicly accessible articles related to the top leading causes of death in the United States—heart disease, stroke, cancer, chronic obstructive pulmonary disease, and diabetes—using three different readability assessment tools: Flesch-Kincaid, simple measure of gobbledygook, and frequency of gobbledygook. They found that the reading levels and comprehension of the articles consistently surpassed the average reading level in the United States, which is between seventh and eighth grade. Leroy et al [20] examined information from WebMD and MEDLINE and reported similar findings, with readability levels above 12th grade. This is also the case for health websites with information written in Spanish [21]. More specifically, research on information finding about breast cancer survivorship shows that overly complicated web-based information can negatively affect patients’ care seeking and treatment decisions [22].

This is problematic, particularly for Hispanics. Hispanic adults in the United States have significantly lower literacy scores when compared with White adults with the same educational level. In terms of reading comprehension, Hispanics scored the lowest of any ethnic group in the United States [23]. When processing web-based information, research shows that, in general, low literacy and low health literacy are detrimental to an individual’s ability to evaluate health information [17].

In general, many researchers state that strategies to increase Hispanics’ access to internet health information will likely help them become empowered and educated consumers, potentially having a favorable impact on health outcomes [24]. However, internet content has changed little to make this possible, and we believe it is important to understand the internet health information search behaviors of Hispanics to effect change.

Given the research cited here, Hispanic survivors of breast cancer fall into a segment of the population that tends to turn away from internet searches. To the best of our knowledge, the present pilot study is the first that does not rely on surveys or interviews to study Hispanics’ health information search behaviors on the web but instead relies on observation.

## Methods

### Recruitment and Study Design

We recruited 7 women from a support group for Hispanic patients and survivors of breast cancer and from a pool of Hispanic women who had participated in other mobile health studies related to cancer education [25]. The women were in remission after diagnosis and treatment of breast cancer. As many of these women rely on their caregivers for information, we asked them to bring their caregivers to the experiment if desired; 43% (3/7) of women brought their caregivers. This resulted in 3 survivor-caregiver dyads and 4 individual survivors.

We asked the participants to talk about their experiences searching for information and the importance of web-based content and search abilities. After this short conversation, we proceeded to have them complete demographic information, the McArthur social mobility ladder [26], and the Health LiTT health literacy questionnaire. This is a short questionnaire that has been used previously in web-based settings with Hispanic women and was designed to address important attributes recommended by the Medical Outcomes Trust for multi-item measures of latent traits [27]. Finally, we asked them to fill out a 16-item breast cancer knowledge questionnaire used in previous mobile health interventions [25]. These questionnaires were given in the language of preference of the participant (English or Spanish).

Following previous research methodology [14], we proceeded to ask them to search for information they thought was relevant on several topics. Each search was given a maximum time of 10 minutes. In addition, we showed participants that they could search using their voice (using Google Chrome and the Google search engine with the option of voice search). For those who preferred to search in Spanish, we configured their search engines to understand Spanish and conducted the whole session in Spanish. After each search, we asked participants to switch to a note-taking application and write a sentence or two about the information they found interesting regarding the topic they were searching.

The first search was free form, and participants were directed to search for any topic they thought was important. We encouraged them to pick topics that may have come up in the questionnaires they had just answered. The purpose of this search was to allow participants to become accustomed to searching on the computers we provided and to switch back and forth from the web browser to the note-taking application. As research suggests that Hispanics prefer voice searches to written ones [15], participants had the chance to search via voice and via text. Once this task was completed and the participants felt comfortable using the computers, voice and written queries, and note-taking applications, the researcher proceeded to ask them to perform three more searches. At this point, the participants could choose whether to use voice searches or written ones. The topics of these additional searches were based on those that are highly correlated with the quality of life of Hispanic survivors of breast cancer [28] and that have come up on surveys and user studies [4]. The topics to search were (1) maintaining good spirits as a survivor of breast cancer, (2) affording treatment and medication, and (3) breast cancer and most common treatments. Consistent with previous research [14], we observed that our first 3 participants were formulating their searches almost verbatim from the researcher’s prompt. Therefore, we added a fourth, more free-form search for the remaining participants: (4) search for any lingering issues they had related to survivorship.

After participants had finished searching, we debriefed and asked about their thoughts regarding their experience searching for these topics. These responses were audio recorded, whereas all computer screen activities were video and audio recorded.

### Analysis

To analyze the participants’ search activities and behaviors, we created a coding scheme with codes divided into four categories. (1) Web activity: in this category, we recorded clicks, clicks on advertisements, images, and clicks on a result. Tracking where users click and the number of clicks it takes a person to find information have traditionally been good indicators of search proficiency and interest in results [29,30]. In this category, we also tracked whether the searches were made by voice or typed as research indicates a different mindset—expectations and behaviors are associated with the modality of search [15]. (2) Participant behavior: here, we recorded whether participants read aloud, whether dyads discussed or talked to each other, or whether there were durations of silence without action; behaviors such as these are mechanisms frequently used by beginner or nonproficient readers for memorization and comprehension [31,32]. (3) Content: under this category, we coded the text of the query, the text of a note, and the webpage URLs they accessed. These allowed for qualitative examination of the search queries and notes taken and allowed us to trace and find information such as the readability level of the websites visited, trustworthiness, and whether they found answers to their queries. Tracking this is important, as readability is important to understand obstacles to low literacy information seekers [33,34], and it can lead to an information *rabbit hole* where users would not find answers to their original query [35]. (4) Information retrieval–related issues: In this category, we tracked misspellings, misappropriations (the wrong word for a given term; for example, lymphomas instead of lymphedema), and speech recognition misunderstandings. All these have been documented as barriers for low-literacy populations when searching on the web [14]. We used the initial free-form searches to train our coders and establish the reliability of the coding scheme. We obtained interrater reliability of k=0.89.

However, because of the small sample size, we mostly report descriptive statistics.

## Results

### Participant Characteristics

The average age of the participants and caregivers was 57.7 (SD 9.9) years. Of the 10 participants, 3 (30%) participants had less than high school education, 2 (20%) had high school diplomas or equivalent, and 5 (50%) had some college education. Of the 10 participants, 3 (30%) were caregivers: 2 (20%) were daughters of the survivor, and 1 (10%) was a friend. None of the participants had a college diploma or higher. In terms of health literacy scores in the Health LiTT test, measured as the proportion of items correct, the minimum score obtained by a participant was 21.4%, the maximum was 50%, and the mean was 37.5% (SD 9.9%). All the women considered themselves Hispanic. Approximately 80% (8/10) of women reported that they felt very comfortable speaking Spanish, and only 40% (4/10) felt very comfortable in English.

In terms of knowledge about breast cancer, in one dyad, the caregiver knew more about breast cancer than the survivor. The average score on the knowledge of breast cancer questionnaire among these women was 66.88%. The 2 caregivers who were daughters of the survivors scored above the mean score, whereas the caregiver who was a friend of the survivor scored very low. Table 1 shows the scores of each participant in the breast cancer knowledge test.

**Table 1:** Breast cancer knowledge scores of survivors and caregivers.

Dyad per participant	Caregiver score, % (relationship to survivor)	Survivor score (%)
1	75 (daughter)	50
2	43.8 (friend)	75
3	N/A^a^	62.5
4	N/A	62.5
5	N/A	93.8
6	N/A	68.8
7	68.8 (daughter)	68.8

^a^N/A: individual participated alone.

### Query Formulation

For each of the searches, we tracked whether they were done via speech (spoken query) or writing (written query). Tables 2-5 show the attempts by our users and whether the search was spoken or written. When a cell has multiple lines, it denotes multiple queries for the same search task. The modality of each query is at the end of each query (S: spoken query; W: written query). The queries in Spanish have their corresponding translations in italics and were provided by one of the researchers. All the original text has been maintained as typed or as transcribed by the speech-to-text Google engine.

**Figure 1:**
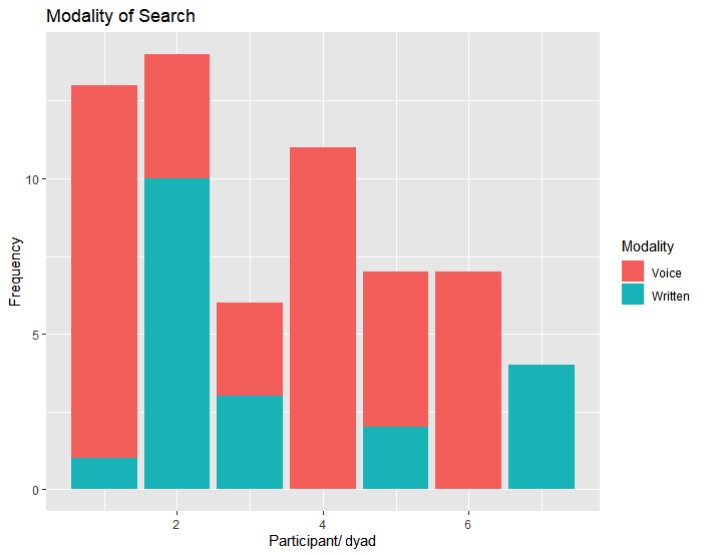
Number of voice versus written search queries per participant per dyad.

As can be seen from these searches, most participants (and dyads) use speech to search for one point or another. In five of the seven sessions, the participants used spoken searches several times before reaching the desired results. Figure 1 shows the number of written searches versus voice searches per participant or dyad.

**Table 2:** Search queries used by participants on the first search.

ID	Searches about breast cancer and most common treatments	Individual or dyad
1	What is breast cancer (S^a^); The whole thing (S); What is breast cancer in the most common treatment (S); What is breast cancer and the most common treatments (W^b^)	Dyad
2	What is breast cancer and better treatment (S)	Dyad
3	Que es el cancer de seno y sus mejores tratamientos? (W; what is breast cancer and its best treatments?)	Individual
4	What is breast cancer and the most common (S) What is breast cancer and the most common treatment(S)	Individual
5	Qué es el cáncer de mama (S; what is breast cancer)	Individual
6	What is breast cancer and the most common treatments (S); What is breast cancer (S)	Individual
7	Cancer de mama y sus tratamientos mas comunes (W; breast cancer and its most common treatments)	Dyad

^a^S: spoken query.

^b^W: written query.

**Table 3:** Search queries used by participants on the second search.

ID	Searches about maintaining good spirits as a breast cancer survivor	Individual or dyad
1	No surviving breast cancer (S^a^); Positive outlook for breast cancer survival (W^b^); How to have a positive attitude about cancer (S); omo mantener el animo positivo (W; [how] to maintain good spirits) Como mantener el animo positivo sobreviviente (W; how to maintain good spirits survivor) Como mantener el animo positivo sobreviviente de cancer de mama (W; how to maintain good spirits survivor breast cancer) Como animar a alguien que tuvo cancer (W; how to cheer up someone who had cancer) Maintaining a positive outlook after cancer (W)	Dyad
2	Breast cancer survivor (S); How to maintain my humor (S); How to maintain a good humor after being a breast cancer survivor (S)	Dyad
3	Cómo mantener un buen ánimo Después (S; how to maintain good spirits after) Cómo mantener el ánimo después del cáncer de seno (S; how to maintain good spirits after breast cancer) Cómo mantener el ánimo después de los del cáncer de seno; (S; how to maintain good spirits after of the breast cancer) Cómo mantener el ánimo después del cáncer de seno (S; how to maintain good spirits after breast cancer)	Individual
4	How to maintain good spirits (S); Here’s a summary from URMC^c^ University (S); How to maintain good spirits as a breast cancer survivor (S)	Individual
5	Cómo tener buen ánimo para cel- (S; how to keep good spirits for cel-[sic]) Cómo es mantener buen ánimo para ser sobreviviente de cáncer de mama (S; how is it to maintain good spirits to be a survivor of breast cancer)	Individual
6	How do I maintain a good spirit after breast cancer (S)	Individual
7	Como mantener buen animo siendo sobreviviente de mama (W; how to maintain good spirits being a breast survivor)	Dyad

^a^S: spoken query.

^b^W: written query.

^c^URMC: University of Rochester Medical Center.

**Table 4:** Search queries used by participants on the third search.

ID	Searches about affording treatment and medication	Individual or dyad
1	How to afford cancer treatment and medication (W^a^) Cancer patient assistance programs (W)	Dyad
2	How could someone (S^b^) Height of someone (S); How could someone afford (S); How can someone afford (S); How can someone afford medication (S); Half of someone afford (S); How can someone afford medication (S); How can someone afford medication cancer and treatment (W)	Dyad
3	Como pagar tratamientos y medicamentos para el cancer de seno? (W; how to afford treatment and medication for breast cancer?)	Individual
4	How can someone afford treatment and medication (S)	Individual
5	Hay organisaciones que ayunan para el tratamiento de mama (W; are there organizations [misspelled] that fast [misspelled help] for the treatment of breast)	Individual
6	Is there an affordable way (S); Is there an affordable way for breast cancer treatment (S)	Individual
7	Que opciones hay para pagar tratamientos del cancer de mama (W; what options are there to afford treatment of breast cancer)	Dyad

^a^W: written query.

^b^S: spoken query.

**Table 5:** Search queries used by participants on the fourth search.

ID	Searches about any lingering issues they had related to survivorship	Individual or dyad
1	N/A^a^	Dyad
2	N/A	Dyad
3	N/A	Individual
4	After 5 years of a breast cancer survivor, can breast cancer come back (S^b^)	Individual
5	Como yo puedo saber que mi cancer no va alvolver (W^c^; how can I know if my cancer is not coming back [misspelled])	Individual
6	After cancer treatment (S); After tonsil cancer treatment (S); Here’s some information for the treatment of tonsil cancer according to cancer research UK (S); Tonsil cancer treatment after surgery combined with chemotherapy (S); Once cancer treatment is over are you considered in (S)	Individual
7	Me podria regresar el cancer (W; could my cancer come back?)	Dyad

^a^N/A: not applicable.

^b^S: spoken query.

^c^W: written query.

We also noticed several search queries that lacked content. For example, “How can someone afford treatment and medication (S)” or “How to maintain good spirits survivor (W).” The results were not necessarily focused on breast cancer because of the lack of context. Moreover, on several occasions, participants clicked to search by voice, and the search engine picked up speech that was not part of the intended search. These included (1) spoken information from a previous result set that Google was still reading when the participants started a new search (eg, “Here’s a summary from URMC University” and “here’s some information for treatment of tonsil cancer according to cancer research UK”); (2) making comments while the computer was listening (eg, “the whole thing”); or (3) not completing their utterance before the search engine started retrieving results, which led to several searches being performed until a query was completely articulated (eg, participant 2 [dyad] in two occasions; Table 4).

Participants reformulated their queries by adding search terms, by switching between spoken or written searches, or, in one case (participant 1 on search 2; Table 3), switching from English to Spanish, trying to obtain adequate results.

It is important to note the behavior of participant or dyad 7. They used only written searches and only one attempt at searching. This was a dyad where the caregiver told us that the survivor was unable to read and trusted her caregiver with finding information. They also arrived late at the experiment and were somewhat constrained by time.

### Readability of Websites Chosen

We kept track of the websites where users obtained information to later take notes they considered important. We submitted the text of the websites in English to a Fleish-Kinkaid analysis. However, Fleisch-Kinkaid does not reflect a correct grade level in Spanish texts, mainly because of the difference in the average number of syllables in a word between English and Spanish. Therefore, for websites in Spanish, we used the Gilliam et al [36] adaptation of the Fry graph for readability (FGR), which has been validated in previous research [21]. As the length and number of sentences are one of the main components of the FGR, we excluded titles and lists that make sentences artificially short and selected a sample of the first two paragraphs of each article in Spanish. The average grade level of the websites in Spanish was 7.15 (SD 0.83). Table 6 shows the websites visited and their average reading grade levels. Websites in Spanish are marked next to their readability grade level. The average is shown separately for the English and Spanish websites.

**Table 6:** Readability scores for websites visited.

Website			Reading grade level
Cancer.net			12.22
Cancer.org			12.08
Komen.org			10.35
Cdc.gov			9.44
Verywellhealth.com			12.51
Lynparzahcp.com			19.28
Ibtimes.com			14.1
Webmd.com			7.78
Chemocare.com			11.2
Prescriptionhope.com			10.56
Cancer.gov			9.07
Mayoclinic.org			8^a^
Roche.com			8^a^
Cancer.gov			6^a^
Vidaysalud.com			7^a^
Cancer.org			7^a^
Unimiamihealth.org			8^a^
Medlineplus.gov			6^a^
Cdc.gov			7^a^
**Overall total, mean (SD)**			
	English	11.33 (3.01)	
	Spanish	7.15 (0.83)^a^	

^a^Websites in Spanish.

The mean grade level readability was 11.33 (SD 3.01) for all the articles in English. All but one of the articles had a readability score below the sixth-grade level. The average readability exceeded the recommended readability (sixth grade) by 5.33 grade levels. Moreover, the readability of the articles exceeded the eighth-grade level by an average of 3.33 grade levels. The only article with readability below the sixth-grade level was one of the articles from WebMD with readability (F-K)=5.8.

The mean grade level readability of the Spanish language articles was 7.15 (SD 0.83), with articles ranging from sixth to eighth grade. This suggests a higher grade level readability for articles in English. Moreover, in one article from cdc.gov, we found an English version and its manual translation into Spanish. The Spanish version from cdc.gov [37] yielded a grade level of seventh grade readability using the Gilliam et al adaptation of the FGR. However, when accessing the translation in English [38], it yielded 14th grade level using the FGR.

### Answers to Participants’ Questions

When analyzing participants’ notes on each search, we found that although many typed pertinent answers, some copied verbatim from snippets in the results lists, resulting in notes with mixed contextual information such as, “Breast cancer is a tumor or mass. Treaty [*sic*] by chemo, mastectomy or Lumpectomy. Radiation therapy.” Others copied and pasted, resulting in notes including URLs and characters that had to do with the formatting of the web pages or notes ending with the start of a new topic: “[...] despues de cinco anos de estar libre de cancer, el viaje aun no termina>> tener los cuidados necesarios, a largo plazo” (“[...] after five years of being cancer free, the journey is not over yet>>having appropriate care in the long term”).

In addition, 2 participants did not find any information with respect to the questions they posed. However, the other participants who visited the same website did. Finally, our coders determined whether the participants answered the questions they posed and found that on eight occasions, the participants did not. Most notably, none of the participants answered their original question on the fourth search, which was free form. For example, participant 4’s question was, “After 5 years of breast cancer survivor, can breast cancer come back,” and her written answer was, “You should keep doing breast self-exams checking the treated area and your other breast exams.”

Satisfactory information was mostly found after clicking, on average, between 1 and 2 websites. However, some users found the information they needed straight from the snippets of text in the results list. In particular, participant 6 never visited a website to answer her questions. Table 7 shows the number of websites visited before the participants wrote their notes to answer their queries.

**Table 7:** Number of websites visited before writing down useful information.

Participant	Search 1^a^	Search 2^b^	Search 3^c^	Search 4^d^
1	2	3	2	N/A^e^
2	1	1	1	N/A
3	4	3	1	N/A
4	2	1	1	1
5	1	0	0	1
6	0	0	0	0
7	0	1	1	1

^a^Search 1: average 1.4 (SD 1.39).

^b^Search 2: average 1.3 (SD 1.25).

^c^Search 3: average 0.9 (SD 0.7).

^d^Search 4: average 0.75 (SD 0.5).

^e^N/A: not applicable.

**Figure 2:**
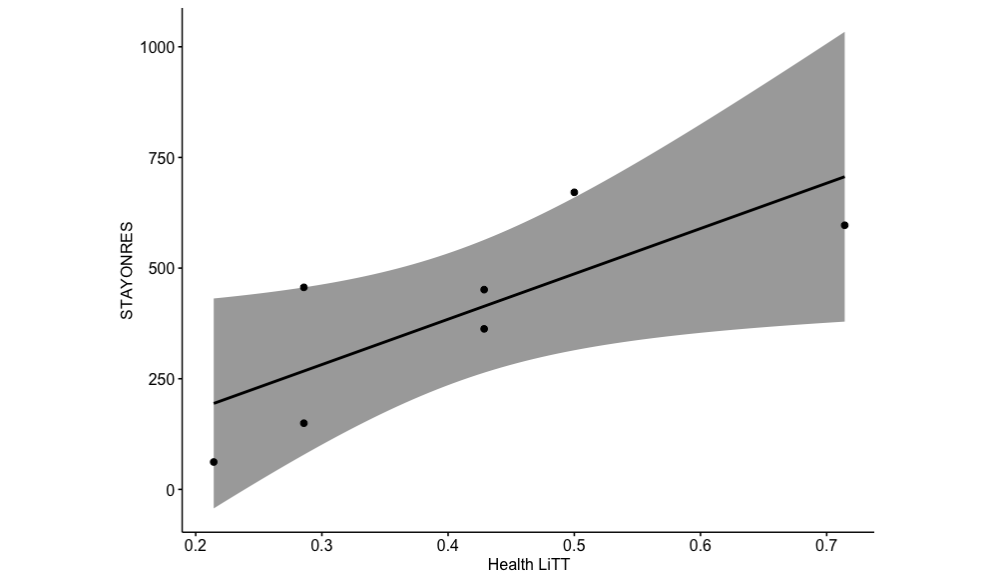
Exploratory correlation of Health LiTT scores as predictors of time (in seconds) spent on individual articles (STAYONRES); R=0.78; *P*=.04. STAYONRES: staying on the results page; Health Litt: Health Literacy Assessment Using Talking Touchscreen Technology.

Our participants spent, on average, 25.5 seconds browsing result lists and 52.9 seconds on actual articles (STAYONRES). However, in 3 of the 7 sessions, participants spent more time browsing the search’s result list than reading information pages. To further explore whether health literacy could be related to the amount of time spent on individual articles, which are comprised of significantly more health-related content than snippets in a list of results, we plotted the Health LiTT scores versus STAYONRES. Figure 2 suggests a potential correlation with this small sample size, which may be worth exploring in future work.

## Discussion

### Principal Findings

In this observational study, we set out to review the search behaviors of Hispanic survivors of breast cancer when examining health-related content. To our knowledge, this study is unique in that it focuses on low-literacy Hispanic survivors of breast cancer and examines searching (1) in their language of preference, (2) using voice as an alternative to writing search queries, and (3) with regard to health literacy and prior knowledge of breast cancer.

None of our participants used isolated search terms (keywords) to formulate queries, as was done in previous studies [14,39]. Instead, they all attempted to use full sentences, whereas on a few occasions inserting the terms *cancer* or *breast cancer* for context. Often, participants searched for a given problem without specifying the context of the search and, thus, obtained less than satisfactory results lists. In particular, and as Birru et al [14] found, search queries were often a verbatim transcription of the prompt the researchers gave the participants. However, in this study, the last search performed was such that the participants needed to find any information interesting to them as naturally as they would search at home. This resulted in mostly fully formulated questions, as opposed to isolated search terms. Perhaps the familiarity with technology has increased in our population to the point that they understand that the interfaces now respond well to full natural language queries.

In terms of results, we found that in agreement with studies from over 15 years ago [21,39], the grade level of the information displayed on the web is far above what most participants are prepared to read.

To find the results, in several instances, participants simply grabbed content from the lists of results. This can be because of their familiarity with their condition and as they may already know some of the answers. However, it can also be as the snippets in the list of results are simpler to read than full-fledged articles, given their low literacy and health literacy levels. This may pose a danger of finding snippets of information without the appropriate context to interpret them adequately. Despite the information being readily available in the articles visited, some participants still struggled to find something useful even when others did. This, again, can be because of a lack of comprehension or novelty of the information. That is, as the patient may already know the basic answers displayed in the results, they may have determined that the information displayed was not useful. It is interesting to note that on the fourth search, which was free form, all patients asked about their cancer coming back but took notes that did not answer the question directly. All searches asked whether cancer could come back. However, their notes were about the continuing care they should take as survivors; that is, their notes were related to the question but not to a direct answer. This could indicate that they have difficulty formulating a question that captures their exact concerns; instead, they ask a more general question hoping to find a detailed answer that resonates with their direct concern. Perhaps their intention was to know how to monitor and prevent the recurrence of breast cancer (which is what all the notes were about).

### Search Modality

Although some participants preferred written searches, most used the spoken search capabilities to a large extent. However, when using the spoken searches, the search engine detected pauses (as to think what to say) as a sign that the query had finished and retrieved results with an incomplete query. For example, “how could someone” was a search term in which the results were not related to breast cancer survivorship. At other times, when faced with a spoken search that retrieved no good results at first, participants wrote the same query hoping that they would get a different results list. However, overall, participants persisted in searching via voice. In conversations after their experiment, they all expressed a desire for it to work and said it was very useful.

### Limitations and Future Work

The main limitation of this study is the number of participants. More participants will certainly add strength to some of our intuitions and may result in strong patterns. For any quantitative analysis to find correlations and significant statistical effects, a power analysis reveals that for the correlations to be ≥0.5, with 80% power and *P*=.05, we would need 18 participants and 18 dyads (to account for correlations of dyads only). A second limitation is that although each participant received training on the use of the computer, technological fluency was not assessed or controlled for. A third limitation is that the notes taken are not necessarily a direct reflection on the comprehension of the texts. Perhaps more subtle measurements of comprehension can be obtained after each search, or we could simulate an urgency to find appropriate results, as this has been documented to enhance and focus on the use of search engines [40]. Finally, not all participants used these kinds of tools on the web (voice search or internet search). Instead, some let their caregivers search on the web and tell them what to do. Therefore, it is important to have an adequate number of caregivers in future research.

## Abbreviations

FGR: Fry graph for readability
